# Study of Protein Biomarkers of Diabetes Mellitus Type 2 and Therapy with Vitamin B1

**DOI:** 10.1155/2015/150176

**Published:** 2015-07-27

**Authors:** Samreen Riaz

**Affiliations:** Department of Microbiology and Molecular Genetics, University of the Punjab, Lahore 54590, Pakistan

## Abstract

In the present research work, the levels of protein biomarkers specific to diabetes mellitus type 2 in the Pakistani population using proteomic technology have been identified and characterized and effect of high dose thiamine has been seen on the levels of these marker proteins. Diabetic patients and normal healthy controls were recruited from the Sheikh Zayed Hospital, Lahore, Pakistan. Total biochemical assays and proteins were estimated by modern proteomic techniques. Some proteins were up- and downregulated in diabetic samples as compared to control and decreased after thiamine therapy, while other protein markers did not show a significant change after the thiamine therapy. The effect of high dose thiamine on the levels of these identified protein biomarkers in the human urine has also been observed. Assessment of the levels of these biomarkers will be helpful in not only early diagnosis but also prognosis of diabetes mellitus type 2.

## 1. Introduction

Diabetes mellitus (DM) is a metabolic and multifactorial syndrome with disordered metabolism and hyperglycemia. On the etiological basis, factors which contributed to the DM and hyperglycemia are reduced secretion of insulin, inherited or acquired insulin deficiency, ineffectiveness of insulin, and low glucose utilization with high production of glucose. The root causes of diabetes are very much complex. Most of the cases start with two processes, metabolic and autoimmune. Some risk factors which contributed to long-term complications for DM are diet, overweight, obesity, smoking, alcohol, level of physical activity, hormones, some medical treatments, viruses, vascular or cardiovascular disease, atherosclerosis, heart conditions, stroke, kidney disease, eye diseases, nerve damage, impaired thinking, infections and wounds, cancer, musculoskeletal disorders, pregnancy complications, emotional difficulties, insulin shock, diabetic ketoacidosis, and hyperosmolar hyperglycemic nonketotic state. The uncontrollable risk factors like genetics and age cannot be altered by people. People can lower the controllable risk factors like exercise and diet through improved health habits and can reduce their risk of developing diabetes [1, 2].

There are several types of DM which exist and are caused by genetic or environmental factors and lifestyle choices. DM is classified into different types on the basis of pathogenic process. The two important types of DM are known as type 1 and type 2. Type 1 DM is juvenile diabetes and is called as insulin dependent diabetes mellitus (IDDM) in which the pancreas fails to produce insulin due to autoimmune beta-cell destruction. It is usually diagnosed in young age like in children, adolescents, and young adults. Type 2 DM is adult-onset diabetes and is called as noninsulin dependent diabetes mellitus (NIDDM) which results from the body tissues and cells inability to respond properly to the action of insulin. There are some other abnormalities like genetic and metabolic abnormalities which are produced in response to insulin action and secretion. Type 2 DM usually occurs in adulthood and develops more with the age and sometimes it is also observed in children and some adolescents having obesity. There are some other types of DM in which specific genetic defects, metabolic and mitochondrial abnormalities, and some conditions that impair glucose tolerance are included [1, 2].

Diabetes is one of the most widely occurring human ailments and the world wide prevalence has risen over the past two decades. According to new publications of some health agencies like World Health Organization (WHO) and International Diabetes Federation (IDF), diabetes becomes an epidemic which is not controlled like other major diseases, for example, cancer and cardiovascular diseases, and becomes sixth leading cause of death worldwide. Humans are not the only species that can develop DM. This disease occurs also in some animals like dogs, cats, and others. Type 2 DM is much more common than type 1 DM and makes up about 90% of all cases of diabetes. It is more common in the developing countries like Pakistan than developed countries. The incidence of this disease in any developed or developing country is difficult to judge. It is quite obvious that the disease is multiplying geometrically more due to genetic and environmental factors [3].

Vitamin is an organic nutrient which is essential and is required in tiny amounts. A vitamin cannot be synthesized by the human body. There are two main types of vitamins: fat soluble and water soluble vitamins. The water soluble vitamins must be eaten more regularly than fat soluble vitamins. Thiamine (vitamin B_1_) is a water soluble vitamin. It has been used singly and in the compound form as a member of B complex family. It has important role in carbohydrates and fat metabolism and is essential for normal growth and development of the human body. It also maintains proper function of the heart, nerves, and digestive system. It occurs as a part of our diet and is present in some diets like cereals, fortified bread, rice, nuts, meats, and legumes. Recommended intake of thiamine for men is 1.4 mg/day, for woman is 1.1 mg/day, for pregnant women is 1.5 mg/day, and for breast feeding is 1.6 mg/day. 100 g corn flakes or 3-4 dL soya milk or 300 g ham covers the daily need. It occurs as a part of our diet. Its deficiency results in a disease called beri beri in which cardiovascular, neurological, and dermatological complications arise. Thiamine deficiency was treated with 50–100 mg of thiamine per day for several days followed by 5–10 mg of oral thiamin per day which was given in parenteral. Treatment is successful in about 50% of patients and replacement of other vitamins as needed. Toxicity of thiamine is not known or reported; it is generally safe. However aside of this restricted use and as a general tonic, it has never been administered as therapy for the diseases for many years [4].

Biomarker is a substance which is used as an indicator for pathological state of disease and a characteristic that is estimated and evaluated for normal, pathological, and pharmacologic responses to a therapeutic intervention. Biomarkers can be used in laboratory for drug discovery, diagnosing, classification, and grading the severity of disease in both laboratory and clinical settings. Biomarkers have a potential for understanding the relationship between disease and health. There are different types of biological markers like protein and genetic and metabolite markers. Biomarkers can also be used for the identification, characterization, and expression of the proteins for biological systems. There are some variations in the patterns of protein expression in normal healthy controls as compared with diseased person. Certain levels of proteins can be up- and downregulated during the progression of disease. Detection of these differences in protein levels is an important area of research in the field of proteomic study. Some of the disease areas of protein biomarkers are, for example, cancer, diabetes, and cardiovascular and neurological diseases. The protein biomarkers are very useful for diagnosis and prognosis of acute and chronic type of mortality in patients having diabetes, various forms of cancer, and other syndromes (Graves and Haystead, 2002).

To identify the protein biomarkers, the proteomic approaches were used that can be exploited for potential diagnostic and prognostic biomarkers in the short and long term of different diseases. Proteomics is the large-scale study of* protein* with their structures, functions, and information coded in a cell which is expressed and regulated at the protein level to achieve the function of an organism. Clinical proteomics aims to discover protein biomarkers which may be a potential target for pharmaceutical field, disease diagnosis, and risk assessment. The goals of proteomics are to apply the proteomic technologies in the clinical research, public health, and environmental, agricultural, and veterinary research [5].

There has been a great interest in the proteomic analysis of plasma and serum for the identification and characterization of protein biomarkers of different diseases. In the modern era for the protein identification, one of the most important developments and technologies is the proteomics. Identification and characterization of low molecular weight proteins in the human plasma/serum and protein profile of human urine have been done by the proteomics. There are many conventional and advanced proteomic technologies that separate the proteins or peptides prior to mass spectrometry (MS) analysis. Ever since O' Ferral introduced the high resolution two-dimensional gel electrophoresis system in 1975, which became the most commonly used technique to analyze protein components of complex mixtures like bacterial extracts and blood plasma. In many ways, the two-dimensional gel electrophoresis was commonly used as a first step for protein identification by mass spectrometry. This approach has made it possible to carry out global protein analysis of living organisms that helps in examining the proteome [6].

Despite considerable reproducibility, this technique could not gain the attention of researchers mainly due to the limitations of this being a labor intensive and qualitative method. Advanced technology in the proteomic filed for the protein separation is the commercial instrument ProteomeLab PF2D from Beckman Coulter. This is the 2D liquid chromatography system (2D-LC) which separates proteins in the first dimension (chromatographic focusing) on the basis of isoelectric points (pI) of protein followed by the second dimension that is reversed phase high performance liquid chromatography (RP-HPLC), where proteins are further fractionated on the basis of hydrophobicity. The assessment, optimization, and separation from the biofluids like plasma, serum, urine, cerebrospinal fluid, and saliva have been observed by the PF2D [7].

**Figure 1:**
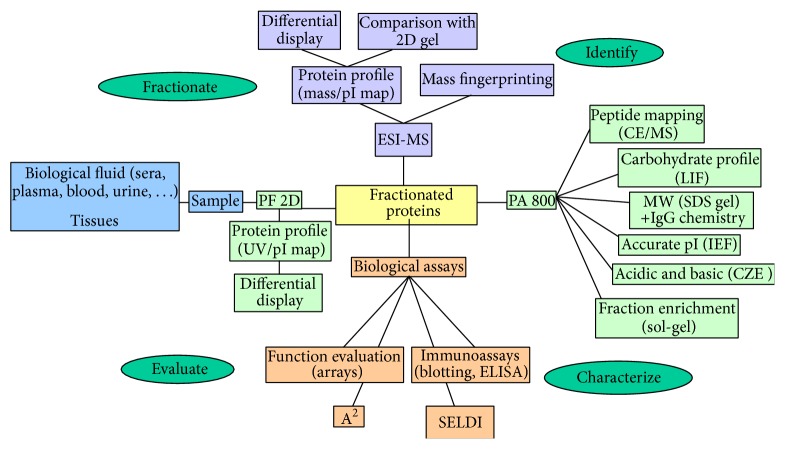
Schematic diagram of protein biomarkers from identification to characterization.

The identification and characterization of protein biomarkers have been achieved by the proteomics coupled with mass spectrometry (MS) analysis. MS technology is highly sensitive and is the most important developments in identification of the proteins. For the detection and characterization of various biomolecules like proteins, peptides, oligosaccharides, and oligonucleotides, the MS technology was used with the molecular mass range between 400 and 350,000 dalton. It detects very low quantities of sample up to 10^−15^ to 10^−18^ mole with an accuracy of 0.1–0.01%. Matrix assistant laser desorption ionization/time of flight (MALDI-TOF MS) is relatively simple to operate. It has good mass accuracy with increased resolution and high sensitivity and is used in the field of proteomics very widely to identify and characterize the protein biomarkers through peptide mass fingerprinting that can be helpful to identify proteins involved in global diseases like diabetes and others. In the method of MS, selected proteins of choice were digested with a specific proteolytic enzyme and the resulting peptides were analyzed for further studies. The information obtained from this can be used for identification and confirmation of the proteins by searching different protein databases. The process of identification and characterization of disease protein biomarkers was summarized and shown in Figure 1 [8].

## 2. Review of the Literature

A large number of people with diabetes especially type 2 grow worldwide. So, this disease takes an ever-increasing proportion of national health care budgets. Without primary prevention, the diabetes epidemic will continue to grow. Immediate action is needed to stem the tide of diabetes. There is also a need of introducing cost-effective treatment strategies to control this epidemic. In the following sections, identification, purification, characterization of protein biomarkers for early diagnosis of pathological states like diabetes mellitus, and role of thiamine on the levels of these marker proteins in diabetes type 2 were described. Research work has been done for the search of protein biomarkers for monitoring and predicting the diabetes mellitus. Several modern techniques mainly involving protein characterization by mass spectrometry and proteomic profiling of plasma/serum samples by proteomics have also been described. Animal studies with high dose thiamine therapy have been shown to reduce diabetic nephropathy (microalbuminuria) and lipid disturbances. Therefore a clinical trial of high dose thiamine therapy has been planned in our type 2 diabetic Pakistani population with a hope to find protein biomarker and role of high dose thiamine on the levels of these biomarkers in the patients having diabetes type 2.

### 2.1. Diabetes Mellitus (DM)

Diabetes results in a condition with abnormal or elevated levels of glucose in the bloodstream. This can cause severe acute and chronic complications like forming brain damage to amputations and heart disease. Dysregulation of multiple glucoregulatory hormones (insulin and glucagons) that maintain glucose homeostasis result in the form of diabetes. The imbalances in these hormones lead to long term elevated levels of glucose and some of microvascular and macrovascular complications like retinopathy, nephropathy, and neuropathy [3].

#### 2.1.1. Regulation of Blood Glucose Level in the Body

**Figure 2:**
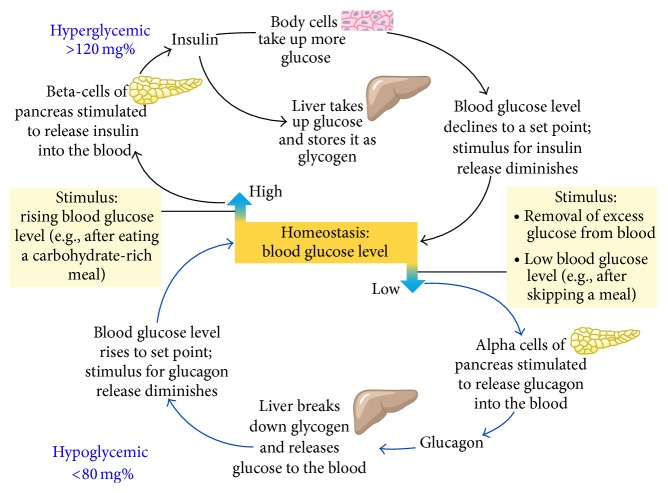
Glucose homeostasis in the human body (adapted from [9]).

The blood sugar at normal levels in the human body is maintained and brought by a regulatory mechanism which is very effective and efficient. In this mechanism, the main organs are liver, autonomic nervous system, and certain glands of internal secretion called endocrine glands. To maintain the homeostasis, the blood glucose is vital for the life of the human being. The important and dominant tissues are liver which respond to the signals that indicate low or high levels of blood glucose. The vital function of liver is to produce glucose for the circulation in the blood. Both low and high levels of blood glucose triggered the hormonal responses which initiate the pathways to restore the glucose homeostasis. Reduced levels of blood glucose trigger release of glucagons from the beta-cells of pancreas, while the increased levels of blood glucose trigger release of insulin from the pancreatic beta-cells. DM results from the dysregulation of multiple glucoregulatory hormones (insulin and glucagons). Any defect in glucoregulatory hormones production leads to improper regulation of glucose in the blood and results in diabetes [9]. This is the glucose homeostasis in which blood glucose levels maintained in the body and dysregulation cause hyper- and hypoglycemia as shown in Figure 2.

#### 2.1.2. Insulin and Its Mechanism of Action in DM

Insulin is a peptide hormone synthesized as precursor of polypeptide preproinsulin with single chain 86 amino acids and is secreted by the beta-cells of the islets of langerhans of pancreatic cells. It maintains the normal levels of glucose in the blood and has mitogenic effects. It facilitates the glucose uptake in the cells, regulates the carbohydrate, lipid, and protein metabolisms, and promotes the cell division and growth. Glucose is the principal stimulus for the secretion of the insulin hormone. Glucose levels >3.9 mmol/L stimulate insulin synthesis. Insulin with its counter regulatory hormone glucagon regulates the concentrations of blood glucose. Beta-cells of pancreases secrete 0.25–1.5 units of insulin per hour during the state of fasting. It is also sufficient to enable the entry of glucose insulin dependent into cells. Any defect in insulin production leads to improper regulation of glucose in the blood and results in diabetes. Similarly, postprandial glucagon secretion is abnormally increased in the patients with type 1 and type 2 diabetes. This abnormal secretion of glucagon leads to excess production of hepatic glucose. It has also played important role in postprandial hyperglycemia in the patients with diabetes mellitus [10].

**Figure 3:**
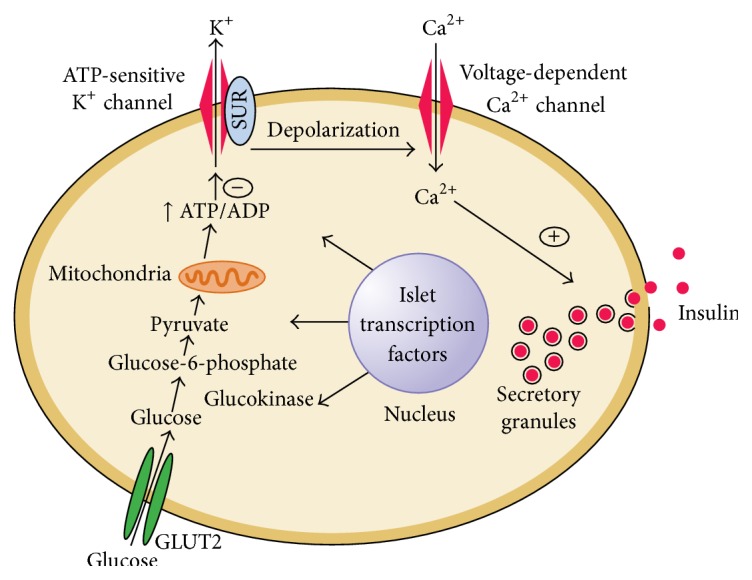
Diabetes and abnormalities in glucose-stimulated insulin secretion (adapted from [9]).

Glucose regulates the insulin secretion by the pancreatic beta-cell through glucose transporters. There are different types of glucose transporters called GLUT1, GLUT2, GLUT3, and GLUT4. Glucose is transported by the glucose transporter GLUT2 in the body. The glucose metabolism in the beta-cells changes the ion channel activity which leads to secretion of insulin. The sulfonylurea receptor (SUR) is the binding site for the drugs that act as secretagogues for the hormone insulin. Glucose stimulation of insulin and its transport also initiate by the GLUT2 into the beta-cell. Phosphorylation of glucose by the enzyme glucokinase controls the insulin secretion regulated by glucose. The metabolism of glucose-6-phosphate via glycolysis produces the ATP which inhibits the activity of an ATP-sensitive potassium channel. Inhibition of this channel initiates the beta-cell membrane depolarization and opens calcium channels that stimulate insulin secretion as shown in Figure 3. Dearrangement in the normal secretary patterns is one of the earliest signs of beta-cell dysfunction in diabetes [9].

Low insulin levels increase the production of glucose by promoting the hepatic gluconeogenesis and glycogenolysis in the fasting state. Glucagons stimulate the glycogenolysis and gluconeogenesis by the liver and the renal medulla. Reduced insulin levels declare the glycogen synthesis and low glucose uptake in insulin sensitive tissues. This also promotes the mobilization of precursors which were stored. Postprandially, glucose load elicits an increase in insulin and decrease in glucagons leading to a reversal of these processes. The main potion of postprandial glucose is utilized by skeletal muscle and insulin stimulated glucose uptake. Other tissues like brain also utilize glucose in an insulin independent manner [11].

#### 2.1.3. Role of Insulin in Type 2 DM

Type 2 DM is a heterogeneous group of disorders and is characterized by the resistance and impaired secretion of insulin and high levels of glucose production. There are some metabolic and genetic defects in the action or secretion of insulin which give rise to hyperglycemia in type 2 DM. Obesity is very common in type 2 diabetes where the adipocytes cells secret a number of biological proteins (leptin, TNF-alpha, resistin, and adiponectin) that modulate insulin secretion or action and may contribute to insulin resistance. The pancreatic islets are unable to sustain the hyperinsulinemia state in the progression of insulin resistance and hyperinsulinemia in certain individuals. Impaired glucose tolerance characterized by elevations in postprandial glucose was developed. Reduced insulin secretion and increased hepatic glucose production lead to hyperglycemia in fasting diabetes. Ultimately the beta-cell failure may increase the release of inflammatory protein markers such as Interleukin-6 (IL-6) and C-reactive protein (CRP) in type 2 DM [11].

**Figure 4:**
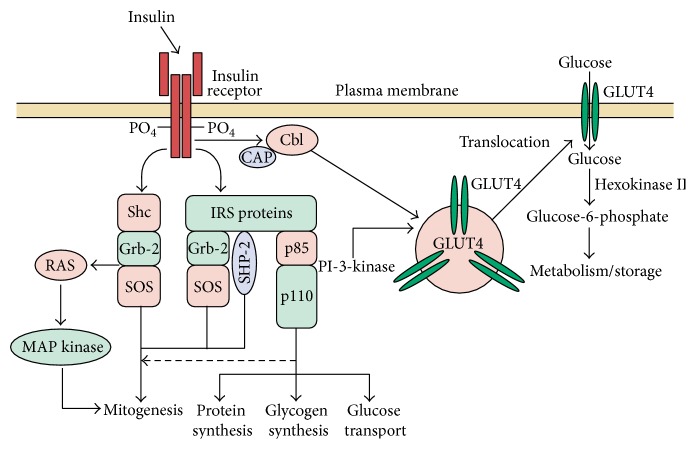
Insulin signal transduction pathway in skeletal muscle (adapted from [9]).

The molecular mechanism of insulin resistance can be explained briefly as levels of insulin receptors and tyrosine kinase activity in skeletal muscles are reduced. These alterations are most likely to cause hyperinsulinemia. Therefore, postreceptor defects play the important and dominant role in insulin resistance. Polymorphism in insulin receptor substrates (IRS) may also be linked with intolerance of glucose, which raised the possibility that polymorphism in different postreceptors molecules combined and created a state of insulin resistance. The pathogenesis of insulin resistance is focused on signaling defect of a phosphatidylinositol-3-kinase (PI-3 kinase). Among other abnormalities, it also lowers the translocation of GLUT4 to the membrane of plasma. The insulin receptor has tyrosine kinase activity which has intrinsic property. These may interact with the proteins of IRS. Many docking proteins bind to these proteins and stimulated the metabolic action of insulin and PI-3-kinase pathway. As a result, insulin gives rise to elevation in glucose transport via PI-3-kinase pathway and stimulates the translocation of intracellular vesicles which has glucose transporters as GLUT4 to the plasma membrane as shown in Figure 4. There are some pathways of insulin signal transduction which are not resistant to effect of insulin like the cell growth/differentiation and mitogenic activated protein (MAP) kinase pathways. As a result, hyperinsulinemia elevated the insulin action through these pathways and accelerated the diabetes and other conditions like atherosclerosis. Free fatty acids can impair the utilization of glucose in the muscles. It also promotes the production of glucose by the liver and impairs the function of beta-cells in the case of obese type 2 DM [9].

### 2.2. Protein Biomarkers for DM

There are varying reports on the role of protein biomarker for the identification of different diseases like cancer, diabetes, and others. There are some proteins which may be up- and downregulated in the serum/plasma and urine of diabetes mellitus especially in type 2.

#### 2.2.1. Identification and Characterization of Protein Biomarkers

The protein biomarkers are very helpful for predicting long-term mortality in patients with diabetes, cancer, and coronary syndromes. Searching for novel biomarkers can be done using tissues and/or biofluids (blood, serum, plasma, and urine). The urine is an ideal biofluid for biomarker discovery in kidney diseases and diabetes mellitus. Urine samples obtained from patients with other diseases or disorders that have clinical, biochemical, and metabolic profiles similar to those of the disease of interest must be included as the other controls. Finally, a single ideal biomarker may not exist for each disease. Therefore, evaluating a panel of multiple biomarkers may be required.

Recently, Thongboonkerd [12] has extensively applied proteomics to biomarker discovery in several diseases with the hope of finding novel biomarkers for earliest diagnosis of the diseases at their very beginning phase and for prediction of therapeutic response, survival, and recurrence. A two-dimensional liquid phase chromatographic separation followed by the mass spectrometry method is involved for proteomics studies and biomarker identification of different diseases. Proteomic analyses using prefractionation strategies were used to identify the biomarker for early detection, diagnosis, prognosis, tumor responses, and disease recurrence for predicting and monitoring biological markers. Proteome analysis of human serum proteins and efficient prefractionation of low abundance proteins in human plasma were done. The construction of two-dimensional map was demonstrated which displayed nearly 3700 chromatographically separated proteins which included 235 more distinct proteins [13]. Complex protein mixture analysis by an all-liquid-phase 2D mapping technique has been done by ProteomeLab PF 2D that is a novel protein profiling and mapping technology. It is also a new powerful way to analyze complex protein mixture in tissues and cells [14]. Detection of electrophoretically derived protein mass based on MS/MS was used as additional constraints in proteomic analysis of human serum [15].

#### 2.2.2. Serum Protein Biomarkers Reported in DM

The proteomics is a valid approach to screen for novel protein biomarkers in animal and human models of obesity and type 2 diabetes. Proteins are reported as biomarkers in the biofluids, tissues, and cells especially in type 2 DM. There are various forms of apolipoproteins which have been identified as protein biomarkers. One of them is apolipoprotein A1 (apoA-I), which is the main component of* high density lipoproteins* (HDL) present in* plasma*. In case of excretion, HDL promotes* cholesterol* efflux from tissues to the liver for excretion (Yui et al., 1998). The level of apoA-1 is positively correlated with HDL cholesterol in the serum and negatively correlated with low density lipoproteins (LDL) cholesterol. Clay et al. [16] have studied the apolipoprotein A-II (ApoA-II) that is in forms of 20% of HDL cholesterol and in human it is present about two-thirds of HDL in humans.

Apolipoprotein H (Apo-H) is also called *β*2-glycoprotein I. It is a plasma glycoprotein present in circulating and free protein or associated with lipoproteins. It has an important role in blood coagulation and clearance of apoptotic bodies from the circulation [17]. The ApoA-I-CIII-AIV levels in type 2 DM and coronary heart disease were studied. The determination of a novel susceptible haplotype was investigated after the study. Apolipoprotein B (Apo-B) is the primary* apolipoprotein* of* LDL*. It is also called bad cholesterol and is responsible for carrying cholesterol to* tissues* [18]. In 2002, Bach-Ngohou and coworkers [19] studied the apolipoprotein E (Apo-E) which is important for the normal* catabolism* of* triglyceride*-rich* lipoproteins*. It is important in lipoprotein metabolism and has a role in* cardiovascular disease*. The serum levels of some protein biomarkers like clusterin and apolipoprotein J were elevated significantly in type 2 DM and during development of coronary heart disease or at myocardial infarction [20]. Nakanishi et al. [21] observed the diabetic macular edema after proteomic analysis of vitreous and recorded six proteins including pigment epithelium derived factor (PEDF), ApoA-4, ApoA-1,* thyroid hormone receptor interactor 11* (Trip-11), retinol binding protein 4 (RBP4), and vitamin D binding protein (VDBP) and only Apo H is present in nondiabetic controls.

Borth in 1992 [22] studied the alpha 2-macroglobulin (*α*2-M) which is an inhibitor for the proteinase enzyme in the blood and tissue. It acts as a binding protein for numerous cytokines and growth factors and also acts as a leptin-binding protein in human plasma. Measurement of levels of protein human *α*_1_-microglobulin (*α*_1_m) was studied in the serum and urine of patients with various liver diseases [23]. Transthyretin and its miracle function and pathogenesis were also observed in one recent research work [24]. In addition, protein biomarker for diabetic nephropathy such as microalbuminuria is also a known predictor for the coronary heart and peripheral vascular diseases and high risk of mortality in patients of type 2 DM [25]. In 1989, Semenkovich and his coworkers [26] observed that the lipoprotein lipase is an important enzyme for lipid homeostasis in humans. It provides intravascular release of fatty acid from circulating triacylglycerol. Lipoprotein lipase is the primary enzyme that converts lipoprotein triglyceride (TG) to free fatty acids. Tumor necrosis factor-*α* (TNF-*α*) is a pleiotropic cytokine and has role in immunity and inflammation and during chronic illness. Its elevated secretion contributed to hemorrhage, necrosis, and death [27]. Leptin was adipokines and has a role in modulating adiposity. This obesity-related hormone is a molecule which regulates the energy balance and body weight [28].

C-reactive protein (CRP) is a protein of acute phase condition and a strong biomarker of inflammation in the progression of various diseases like coronary heart disease, cancer, diabetes, and others. Genetic variation and levels of CRP and incidence of diabetes especially risk of developing type 2 diabetes mellitus have been studies. The concentration of CRP in the blood of normal healthy control human beings ranges from 0 to 1.0 mg/dL. In the acute phase condition of inflammation the CRP levels may rise up to the 1000-fold [29]. In 2002, Lindsay and coworkers [30] demonstrated that some of the proteins are downregulated in DM likeone of the adipokines adiponectin in Indian population. It is a collagen-like plasma protein produced and secreted by adipose tissue. It has compelling antiatherogenic and insulin-sensitizing properties.

Few years later, Martín-Gallán et al. [31] studied that some protein biomarkers of diabetes are associated in young diabetic patients with oxidative stress and antioxidant status. Proteomic analysis of proteins was done and involved in insulin resistance and type 2 DM with the b3-Adrenergic receptor [32]. Proteomics detects oxidatively induced protein carbonyls in muscles of a diabetic rat. A number of proteins, including mitochondrial ATP synthase, desmin, actin, and myosin, are found carbonylated [33]. Festa et al. 2002 [34] have described that the elevated levels of acute-phase proteins and plasminogen activator inhibitor-1 (PAI-1) in type 2 diabetes with insulin resistance were observed. In type 2 DM, the intramuscular heat shock protein 72 and heme oxygenase-1 mRNA were decreased [35]. In literature, diabetes is associated with pancreatic cancer in 80% cases. Tumor-derived peptide with an S-calcium binding protein is involved in the pancreatic cancer associated with diabetes which are the studies of Bassoa and coworkers [36]. Lipids and lipoproteins have been studied in patients with type 2 DM. Recently, obesity and its related proteins like leptin were studied in the diabetic population of Pakistan [37]. List of identified proteins in human serum of patients having diabetes mellitus is given in Table 1.

#### 2.2.3. Urinary Protein Biomarkers Reported in DM

Urinary protein profiling can reveal changes in excretion rates of specific proteins that can have predictive value in the clinical arena, for example, in the early diagnosis of disease, classification of disease with regard to likely therapeutic responses, assessment of prognosis, and monitoring response to therapy.

Yang et al. in 2005 [38] have described that RBP4 protein is an important biomarker for insulin resistance in diabetes. In one such study which was carried out by Metz and coworkers [39], the five protein biomarkers were identified as 2-fold upregulated in diabetes which were alpha-2-glycoprotein (zinc), corticosteroid-binding globulin, and lumican. The clusterin and serotransferrin were 2-fold downregulated in diabetic samples compared to control. For urinary biomarkers in diabetic nephropathy, proteomic analyses identified some potential protein biomarkers.

List of urinary proteins that are identified in diabetic patients with or without nephropathy are listed in Table 2.

In 2007, Rao et al. have observed that there were seven proteins which were upregulated with increasing albuminuria and four proteins were downregulated.

After some years, the proteomic analysis was done for the identification of human salivary biomarkers for type-2 diabetes [24]. Recently, in 2009, Jiang and his coworkers [40] studied the identification of urinary soluble E-cadherin as a novel biomarker for diabetic nephropathy. Shinton et al. [41] observed that haptoglobins are a group of serum proteins; originally identified and diagnostic values were determined.

The goals of present research work were the identification and characterization of protein biomarkers for early diagnosis and prognosis of pathological states of diabetes mellitus type 2 and effects of high dose thiamine on the levels of marker proteins. These investigations shall be helpful in assessing the biochemical alterations in the Pakistani diabetic population, which should contribute to the development of treatment plans for this disease which is one of the most widely occurring and debilitating diseases. Results from this research will also contribute to the identification of protein markers of diabetes mellitus type 2 and thus development of novel diagnostic procedures for early detection of this complication in potential diabetics in our population. The findings from present research work will assist in planning preventive and effective treatment strategies for diabetic patients by identification of protein biomarkers and high dose thiamine as nutritional supplement.

## Figures and Tables

**Table 1:** Serum proteins identified in diabetes mellitus.

Serial number	Category of protein	Name of protein	Expression in diabetics
1	Cytokines and cytokine-related proteins	Leptin	+
		TNF-alpha	+
		IL-6	+

2	Other immune-related proteins	MCP-1	+

3	Proteins involved in fibrinolytic system	PAI-1	+
		Tissue factor	+

4	Complement and complement-related proteins	Adipsin (complement factor D)	+
		ASP	+
		Adiponectin	−

5	Lipids and proteins for lipid metabolism or transport	Lipoprotein lipase (LPL)	+
		Apolipoprotein E	+
		Apolipoprotein A1,	−
		Apolipoprotein A2	−
		Apolipoprotein B	+
		Apolipoprotein H	−
		Apolipoprotein C1, C2	−
		NEFAs	+
		Cholesterol ester transferase protein (CETP)	+

6	Inflammatory proteins	C-reactive protein (CRP)	+
		*α*-tumor necrosis factor (*α*TNF)	+

**Table 2:** Urinary protein identified in diabetes mellitus.

Serial number	Category of protein	Name of protein	Expression in diabetics
1	Defense response	*α*1-antitrypsin Complement factor H, C3, B, I, C7, 9 Alpha-1-antichymotrypsin precursor Antithrombin-III Alpha-2-glycoprotein 1, zinc Ig gama 1 chain C region Alpha and beta-2-microglobulin Alpha-2-antiplasmin precursor Vitronectin precursor	+ + + + + + + + +

2	Transport	Serotransferrin precursor Ceruloplasmin precursor Hemopexin AMBP protein Albumin Haptoglobin precursor Transthyretin precursor VDBP	− + + + − + − −

3	Metabolism	ApoA-1, ApoA-II precursor, Apo-D Alpha-1B-glycoprotein Beta-2-glycoprotein 1 precursor Prostaglandin H2 D-isomerase precursor Alpha-2-HS-glycoprotein precursor E-cadherin Dystroglycan precursor Fibrinogen beta chain precursor	− + + + + + + +

4	Signal transduction	Kininogen precursor B-factor, properdin Clusterin Angiotensinogen Sulfated glycoprotein 2 retinol-binding protein 4 Epidermal growth factor	+ + − + + + +

5	Cell development	Lumican precursor	+

## References

[1] American Diabetes Association (2007). Standards of medical care in diabetes—2007. *Diabetes Care*.

[2] American Diabetes Association (2007). Diagnosis and classification of diabetes mellitus. *Diabetes Care*.

[3] Riaz S. (2009). Diabetes mellitus. *Scientific Research and Essays*.

[4] WHO (1967). *Requirements of Vitamin A, Thiamine, Riboflavine and Niacin. Report of a Joint FAO/WHO Expert Group*.

[5] Frank R., Hargreaves R. (2003). Clinical biomarkers in drug discovery and development. *Nature Reviews Drug Discovery*.

[6] Ferber G. (2002). Biomarkers and proof of concept. *Methods and Findings in Experimental and Clinical Pharmacology*.

[7] Betgovargez E., Simonian M. H. (2003). Reproducibility and dynamic range characteristics of the ProteomeLab PF2D system. *Beckman Coulter Application Information Bulletin*.

[8] Aebersold R., Mann M. (2003). Mass spectrometry-based proteomics. *Nature*.

[9] Lowe W. L. (2005). *Principles of Molecular Medicine*.

[10] Brownlee M. (2001). Biochemistry and molecular cell biology of diabetic complications. *Nature*.

[11] Himsworth H. P. (1936). Diabetes mellitus: its differentiation into insulin-sensitive and insulin-insensitive types. *The Lancet*.

[12] Thongboonkerd V. (2010). Current status of renal and urinary proteomics: ready for routine clinical application. *Nephrology Dialysis Transplantation*.

[13] Pieper R., Gatlin C. L., Makusky A. J. (2003). The human serum proteome: display of nearly 3700 chromatographically separated protein spots on two-dimensional electrophoresis gels and identification of 325 distinct proteins. *Proteomics*.

[14] Simonian M. H., Betgovargez E. (2005). *Proteome Analysis of Human Plasma with ProteomeLab PF2D System*.

[15] Kim J. Y., Lee J. H., Park G. W. (2005). Utility of electrophoretically derived protein mass estimates as additional constraints in proteome analysis of human serum based on MS/MS analysis. *Proteomics*.

[16] Clay M. A., Cehic D. A., Pyle D. H., Rye K.-A., Barter P. J. (1999). Formation of apolipoprotein-specific high-density lipoprotein particles from lipid-free apolipoproteins A-I and A-II. *Biochemical Journal*.

[17] Wang F., Xia O.-F., Sui S.-F. (2002). Human apolipoprotein H may have various orientations when attached to lipid layer. *Biophysical Journal*.

[18] Richardson P. E., Manchekar M., Dashti N. (2005). Assembly of lipoprotein particles containing apolipoprotein-B: structural model for the nascent lipoprotein particle. *Biophysical Journal*.

[19] Bach-Ngohou K., Ouguerram K., Nazih H. (2002). Apolipoprotein E kinetics: influence of insulin resistance and type 2 diabetes. *International Journal of Obesity*.

[20] Trougakos I. P., Poulakou M., Stathatos M., Chalikia A., Melidonis A., Gonos E. S. (2002). Serum levels of the senescence biomarker clusterin/apolipoprotein J increase significantly in diabetes type II and during development of coronary heart disease or at myocardial infarction. *Experimental Gerontology*.

[21] Nakanishi T., Koyama R., Ikeda T., Shimizu A. (2002). Catalogue of soluble proteins in the human vitreous humor: comparison between diabetic retinopathy and macular hole. *Journal of Chromatography B: Analytical Technologies in the Biomedical and Life Sciences*.

[22] Borth W. (1992). *α*2-macroglobulin, a multifunctional binding protein with targeting characteristics. *The FASEB Journal*.

[23] Itoh Y., Enomoto H., Takagi K., Kawai T., Yamanaka T. (1983). Human alpha1-microglobulin in various hepatic disorders. *Digestion*.

[24] Rao P. V., Reddy A., Lu X. (2009). Transthyretin and it's miracle function and pathogenesis. *Journal of Proteome Research*.

[25] James S. K., Lindahl B., Timmer J. R. (2006). Usefulness of biomarkers for predicting long-term mortality in patients with diabetes mellitus and non-ST-elevation acute coronary syndromes (a GUSTO IV substudy). *The American Journal of Cardiology*.

[26] Semenkovich C. F., Chen S.-H., Wims M., Luo C.-C., Li W.-H., Chan L. (1989). Lipoprotein lipase and hepatic lipase mRNA tissue specific expression, developmental regulation, and evolution. *Journal of Lipid Research*.

[27] Graves D. T., Cochran D. (2003). The contribution of interleukin-1 and tumor necrosis factor to periodontal tissue destruction. *Journal of Periodontology*.

[28] Campfield L. A., Smith F. J., Burn P. (1996). The OB protein (leptin) pathway—a link between adipose tissue mass and central neural networks. *Hormone and Metabolic Research*.

[29] Myles D. A. A., Rule S. A., DeLucas L. J. (1990). Rotation function studies of human C-reactive protein. Lipid, Lipoproteins, C-Reactive Protein, and Hemostatic Factors at Baseline in the Diabetes Prevention Program. *Journal of Molecular Biology*.

[30] Lindsay R. S., Funahashi T., Hanson R. L. (2002). Adiponectin and development of type 2 diabetes in the Pima Indian population. *The Lancet*.

[31] Martín-Gallán P., Carrascosa A., Gussinyé M., Domínguez C. (2003). Biomarkers of diabetes-associated oxidative stress and antioxidant status in young diabetic patients with or without subclinical complications. *Free Radical Biology and Medicine*.

[32] García-Rubi E., Calles-Escandón J. (1999). Insulin resistance and type 2 diabetes mellitus: its relationship with the *β*
_3_-adrenergic receptor. *Archives of Medical Research*.

[33] Oh-Ishi M., Ueno T., Maeda T. (2003). Proteomic method detects oxidatively induced protein carbonyls in muscles of a diabetes model Otsuka Long-Evans Tokushima Fatty (OLETF) rat. *Free Radical Biology and Medicine*.

[34] Festa A., D'Agostino R., Tracy R. P., Haffner S. M. (2002). Elevated levels of acute-phase proteins and plasminogen activator inhibitor-1 predict the development of type 2 diabetes: the insulin resistance atherosclerosis study. *Diabetes*.

[35] Mark A. F. (2003). Intramuscular heat shock protein 72 and heme oxygenase-1 mRNA are reduced in patients with type 2 diabetes: evidence that insulin resistance is associated with a disturbed antioxidant defense mechanism. *Diabetes*.

[36] Bassoa D., Grecoa E., Fogara P., Puccib P., Flagiellob A., Plebania M. (2005). Pancreatic cancer-associated diabetes mellitus: an open field for proteomic applications. *Clinica Chimica Acta*.

[37] Riaz S., Alam S. S., Raza M., Hasnain S., Akhtar M. W. (2009). Obesity as risk factor and study of obesity related proteins in diabetes mellitus. *African Journal of Biotechnology*.

[38] Yang Q., Graham T. E., Mody N. (2005). Serum retinol binding protein 4 contributes to insulin resistance in obesity and type 2 diabetes. *Nature*.

[39] Metz T. O., Qian W.-J., Jacobs J. M. (2008). Application of proteomics in the discovery of candidate protein biomarkers in a diabetes autoantibody standardization program sample subset. *Journal of Proteome Research*.

[40] Jiang H., Guan G., Zhang R. (2009). Identification of urinary soluble E-cadherin as a novel biomarker for diabetic nephropathy. *Diabetes/Metabolism Research and Reviews*.

[41] Shinton N. K., Richradson R. W., Williams J. D. F. (1965). Diagnostic value of serum haptoglobin. *Journal of Clinical Pathology*.

